# Crosstalk Between Mammalian Antiviral Pathways

**DOI:** 10.3390/ncrna5010029

**Published:** 2019-03-22

**Authors:** Samir F. Watson, Lisanne I. Knol, Jeroen Witteveldt, Sara Macias

**Affiliations:** Institute of Immunology and Infection Research, School of Biological Sciences, University of Edinburgh, Edinburgh EH9 3FL, UK; s.f.watson@sms.ed.ac.uk (S.F.W.); s1584602@sms.ed.ac.uk (L.I.K.); jeroen.witteveldt@ed.ac.uk (J.W.)

**Keywords:** interferon, antiviral, RNAi, virus, miRNAs, Drosha, Dicer, dsRNA

## Abstract

As part of their innate immune response against viral infections, mammals activate the expression of type I interferons to prevent viral replication and dissemination. An antiviral RNAi-based response can be also activated in mammals, suggesting that several mechanisms can co-occur in the same cell and that these pathways must interact to enable the best antiviral response. Here, we will review how the classical type I interferon response and the recently described antiviral RNAi pathways interact in mammalian cells. Specifically, we will uncover how the small RNA biogenesis pathway, composed by the nucleases Drosha and Dicer can act as direct antiviral factors, and how the type-I interferon response regulates the function of these. We will also describe how the factors involved in small RNA biogenesis and specific small RNAs impact the activation of the type I interferon response and antiviral activity. With this, we aim to expose the complex and intricate network of interactions between the different antiviral pathways in mammals.

## 1. Introduction

### 1.1. The Mammalian Type-I IFN Response

Interferons (IFNs) are the major cytokines expressed during the innate immune response against invading pathogens, such as viruses, bacteria, fungi, and parasites. Besides their role in restricting infections, IFNs also exhibit immunomodulatory functions, and have been implicated in both cancer immunosurveillance and autoimmunity [1,2,3]. IFNs can be produced by virtually all nucleated cells of jawed vertebrates and are classified into three major types: Type I, Type II, and Type III IFNs [4,5]. Type I IFNs are the most diverse family with 8 known subtypes, of which IFN-α and IFN-β can be expressed by nearly every cell type [6,7]. Type II IFN has only one member, IFN-γ, and is mainly expressed by activated natural killer (NK) and T-cells [8,9,10]. Type III IFNs, IFN-λs, have only been recently discovered and are also expressed in multiple cell types [11,12,13,14]. In this review we will focus on the roles of type I IFNs as they are crucial, ubiquitously expressed components of the antiviral response in mammals. 

In the context of viral infections, the type I IFN response is activated by sensing the presence of invading viruses. These pathogens pose a particular challenge for detection by the innate immune system due to their small size and constantly evolving surface protein repertoire, therefore, host cells have developed the ability to recognize virus-specific nucleic acid signatures. These sensing mechanisms rely on host proteins termed pattern recognition receptors (PRRs) recognizing specific pathogen features, known as pathogen-associated molecular patterns (PAMPs). During viral infections, PRRs need to appropriately discriminate between non-self, viral-derived nucleic acids and self-derived nucleic acids (Table 1) [15,16]. The binding and recognition of virus-specific nucleic acids by PRRs is necessary for the production and secretion of type I IFNs and pro-inflammatory cytokines. Secreted type I IFN proteins act in an auto- and paracrine fashion by binding to the heterodimeric type I IFN receptors, IFNAR1 and 2, on the surface of the infected and neighboring cells. This initiates the janus kinase (JAK)/signal transducer and activator of transcription (STAT) signaling pathway that activates a second transcriptional response of around 500 IFN-stimulated genes (ISGs), which establishes the antiviral state [17,18]. These processes constitute the first steps of the innate immune response to infections prior to activation of the adaptive immune system. Here we focus on the mechanisms that cells use to detect invasion by viruses. 

### 1.2. Viral-Derived Nucleic Acids Sensing Mechanisms

#### 1.2.1. TLR-Detection of Viral Nucleic Acids

Toll-like receptors (TLRs) are transmembrane proteins that are located on the cell surface and endosomes. The expression of TLRs is cell specific and each is specialized in the recognition of specific viral-derived PAMPs [19,20]. With the exception of TLR3, which is specialized in dsRNA recognition [21], TLR signaling depends on the adaptor protein myeloid differentiation primary response 88 (MyD88) [20]. MyD88 activates the MAPK pathway, NFκB, and IRF7 transcription factors to induce expression of type I IFNs and pro-inflammatory cytokines [22,23]. TLR3, on the other hand, signals through TRIF to activate the MAPK, NFκB, and IRF3 pathways to induce the expression of similar cytokines [24].

#### 1.2.2. RLR-Detection of Virus-Derived RNA

The presence of cytoplasmic virus-derived RNA is detected by a family of receptors called RIG-I-like receptors (RLRs), which include retinoic-acid-inducible protein 1 (RIG-I), melanoma-differentiation-associated gene 5 (MDA5), laboratory of genetics physiology 2 (LGP2), and signals through the mitochondrial antiviral-signaling protein (MAVS). Activation of RLR-signaling by recognition of virus-derived RNAs also results in type I IFNs and proinflammatory cytokine expression and this, in turn, increases expression of RLRs since these are also ISGs [25,26]. 

**Figure 1:**
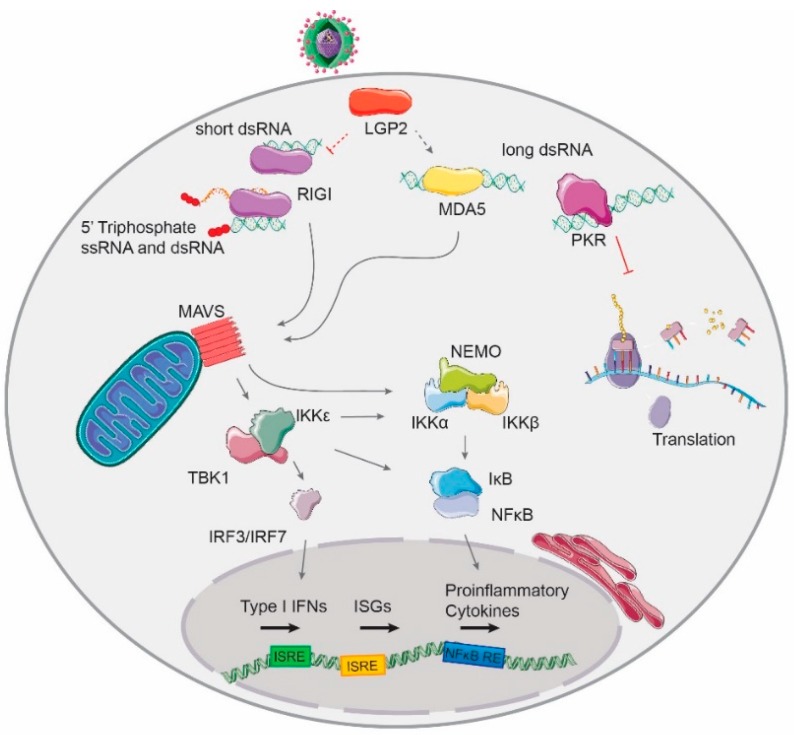
The typical hallmark of viral replication, dsRNA, is recognized by the RLR family of receptors in the cytoplasm of infected cells. RIG-I recognizes short dsRNA molecules with 5′tri- and diphosphates; MDA5 recognizes long dsRNA molecules. Both MDA5 and RIG-I signal through the mitochondrial-associated factor MAVS to activate the transcription of type I interferons (IFNs) and pro-inflammatory cytokines. The third member of the RLR family, LGP2, modulates the activation of RIG-I and MDA5-mediated signaling pathways. The presence of dsRNA activates the host translational shut-off response through phosphorylation of the translation factor eIF2α by the kinase PKR.

RIG-I and MDA5 are structurally similar, consisting of a central DExD/H RNA helicase domain, two tandem caspase activation and recruitment domains (CARD) at the N-terminal end and a regulatory C-terminal regulatory domain [25]. RIG-I and MDA5 bind a complementary set of viral RNA ligands: MDA5 binds long dsRNA, whereas RIG-I binds 5′tri- and diphosphate short dsRNA [27,28,29]. Binding of ligands occurs at the basic cleft in their C-terminal domains (CTD) which leads to a conformational change to expose the occluded CARD domain [30,31]. This causes RIG-I to form tetramers and short filaments and MDA5 to oligomerize and form long filaments along the length of the dsRNA [32,33]. After filament formation, the CARD domains of both MDA5 and RIG-I interact with the CARD domain of MAVS to promote further signaling [34,35]. MAVS activation causes NF-kB, IRF3, and IRF7 transcription factors to translocate to the nucleus and initiate type I IFNs and pro-inflammatory cytokines expression [36,37] (Figure 1). LGP2, which lacks a CARD domain, seems to regulate both RIG-I and MDA5 activity. LGP2 can enhance the rate of nucleation and consequent filament formation by MDA5 [38,39], whereas it inhibits RIG-I-mediated signaling by competing for the same RNA substrates [40]. Thus, the third RLR can act both as a positive or negative factor for antiviral signaling [41,42,43].

#### 1.2.3. Other Antiviral dsRNA-Activated Pathways

**PKR.** Viral-derived dsRNA can also be sensed in the cytoplasm by the dsRNA-binding protein kinase PKR [44]. Upon binding to dsRNA, PKR phosphorylates eIF2α at Ser51 causing the sequestration of the guanine nucleotide exchange factor eIF2B which results in cap-dependent translation inhibition [45,46,47]. This process is also known as the host translational shutoff response (Figure 1).

**OAS/RNaseL.** The presence of cytoplasmic virus-derived dsRNA also activates the OAS/RNase L degradation pathway. The OAS (oligo-adenylate synthase) proteins synthesize 2′,5′-linked adenylates upon binding to dsRNA [48]. These oligomers activate the endoribonuclease RNAse L to cleave ssRNA (single-stranded) in a non-sequence specific manner, preventing viral replication [49,50,51]. RNAse L amplifies IFN signaling further through the production of small RNA cleavage products that can activate RIG-I and MDA5-mediated responses [52]. 

**ADAR**. Specific isoforms of the dsRNA binding proteins of the ADAR family have also been implicated in the regulation of the IFN response and are ISGs. ADAR1 and 2 have been shown to prevent activation of the Interferon response by guiding deamination of adenosines to inosines on endogenous dsRNAs. Unlike unmodified dsRNAs, the presence of inosine residues prevents activation of the innate immune response, while still being bound to RLRs. Consequently, the absence of deaminase activity inhibits the cell’s ability to discriminate “self” from “non-self” and results in the undesired activation of a type I IFN response [53,54]. Besides its importance in regulating the IFN response, ADAR proteins have been shown to directly target viral RNA, resulting in pro- and antiviral effects [55].

Additional dsRNA binding factors, such as Drosha and Dicer, classically involved in the biogenesis of small RNAs, have been recently found to provide alternative antiviral activity independent of the IFN response, which will be discussed below.

### 1.3. Mammalian Small RNA Biogenesis

Mammalian endogenous small RNAs (20–30 nt long) can be divided into three different categories depending on their biogenesis pathway: micro (mi)RNAs, short-interfering (si)RNAs, and PIWI-interacting (pi)RNAs [56]. mi- and siRNAs associate with Argonaute (Ago) proteins to guide post-transcriptional regulation of target mRNAs expression, whereas piRNAs associate with a subfamily of Ago, PIWI proteins, to target transposable elements in the germline [57]. 

**Figure 2:**
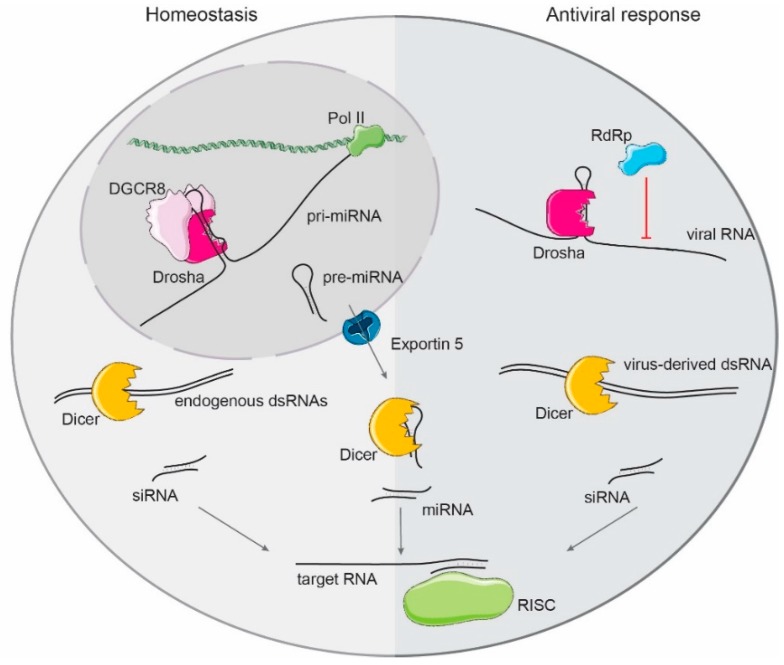
Primary miRNA (pri-miRNA) precursors are transcribed by RNA-polymerase II, and processed in the nucleus by the microprocessor complex, composed by DGCR8 and Drosha. The released hairpin (pre-miRNA) is exported to the cytoplasm by Exportin-5 to be further processed by Dicer to form mature miRNAs that are loaded into the RISC complex to target complementary mRNAs. Both Drosha and Dicer are also antiviral factors. Drosha binding to RNA secondary structures in the viral genomes blocks the viral RNA-dependent RNA-polymerase and replication of the virus. Dicer can cleave the virus-derived dsRNA intermediates of replication to generate small interfering RNAs (siRNAs) that target and induce the decay of viral RNA molecules.

miRNAs are transcribed in the nucleus by RNA-polymerase II as long precursor molecules (pri-miRNAs) that can be organized in clusters, indicating that a single transcript is sufficient to produce different miRNAs [58,59]. Pri-miRNAs adopt stem-loop structures that are recognized and cleaved by the nuclear microprocessor complex, which consists of the RNAse III endonuclease Drosha associated with two copies of the dsRNA binding protein DiGeorge syndrome chromosomal region 8 (DGCR8) [60,61,62,63,64,65] (Figure 2). DGCR8, interacting with the apical part of the pri-miRNA hairpin, guides Drosha binding and cleavage at the base of the hairpin, releasing a ~60–70 nt hairpin with a 2-nucleotide 3′ overhang [62,65,66,67]. Besides the typical RNA secondary structure of pri-miRNAs, specific sequence motifs determine successful processing. Efficiently processed pri-miRNAs harbor a 5′ UG motif at the basal junction, a UGU motif at the apical loop, a mismatched GHG motif in the 3′ stem region, and a 3′ flanking CNNC motif [65,66,68,69]. Microprocessor processing efficiency is also regulated by additional auxiliary factors that enhance or inhibit its cleavage activity on specific pri-miRNAs [70]. After microprocessor processing, the cleaved hairpin structure (pre-miRNA) is transported to the cytoplasm by Exportin 5 [71,72], where further processing by the RNAse III endonuclease Dicer takes place. Cleavage of pre-miRNAs by Dicer produces the mature miRNA duplex of 20–24 nt length, containing 2-nucleotides 3′ overhangs on both strands [73,74,75,76]. Only one strand of this duplex, the guide strand, is loaded into the RNA-induced silencing complex (RISC) to inhibit expression of the target mRNA [77,78,79] (Figure 2). In mammals, four different Ago proteins are expressed and associate with miRNAs (Ago 1–4) [80,81], of which only Ago2 and Ago3 have retained endonuclease activity [82,83]. The additional co-factors TRBP and PACT in humans, and Loquacious (Loqs) in *D. melanogaster*, are necessary for successful Dicer-mediated miRNA processing and formation of the RISC complex [84,85,86,87,88,89].

The binding of the mature miRNA to the target is dependent on partial complementarity to a short (6–7 nt long) sequence in the miRNA, known as the seed sequence, which is enough to mediate translational repression or destabilization of the bound mRNAs [90,91,92,93].

In addition to the canonical miRNA biogenesis pathway, several miRNAs have evolved to use alternative biogenesis routes. Mirtrons are microprocessor-independent miRNAs derived from spliced introns, which are processed by Dicer in the cytoplasm [94,95,96]. Specific miRNAs, such as mir-451, are processed by the microprocessor, followed by an Ago-2 dependent processing step [97,98,99]. Other Ago-associated RNAs, termed Agotrons, bypass both the microprocessor and Dicer processing steps [100,101].

Apart from its role in miRNA biogenesis, Dicer is essential for the production of siRNAs in chordates and non-chordates (Figure 2). Cytoplasmic processing of endogenous dsRNAs derived from sense and antisense transcripts or long stem-loop structures by Dicer generates mature siRNAs that are also loaded into the RISC complex [102,103,104]. Unlike miRNAs, the full complementarity between siRNAs and the target activates Ago2 endonucleolytic activity and degradation of the target RNA [82,83,105]. In mammals, endogenous siRNAs (endo-siRNAs) have been reported in mouse embryonic stem cells and oocytes. In stem cells, endo-siRNAs originate from repetitive elements (SINEs, short interspersed elements) [102], and in oocytes, endo-siRNAs have been shown to control the expression of both mRNAs and retrotransposons [106,107]. In comparison to non-vertebrate organisms such as *C. elegans* [108,109,110] or *D. melanogaster* [103,104,111], it is still unclear how widespread the synthesis and function of endo-siRNAs in mammals is. Initially, mammals were not considered to produce siRNAs, since the presence of dsRNAs in the cytoplasm could trigger a type I IFN response. Noticeably, the cellular models where mammalian endo-siRNAs have been reported have an inherently attenuated IFN response [112,113,114,115].

Interestingly, both Drosha and Dicer have been recently shown to have a direct role in controlling viral infections, apart from their classical function in small RNA biogenesis (Figure 2).

## 2. Drosha and Dicer as Direct Antiviral Factors

### 2.1. Drosha

The microprocessor complex can process other cellular RNAs adopting structures resembling pri-miRNAs outside the canonical substrates [116,117,118]. For example, cleavage of hairpin structures contained in the 5′UTR of DGCR8 mRNA downregulates Dgcr8 expression and consequently microprocessor function, a mechanism conserved in humans, mice, and flies [119,120,121]. Other non-canonical microprocessor functions include the control of transposable elements, where hairpins contained in the autonomous LINE-1 and non-autonomous Alu retrotransposons are cleaved by the microprocessor to regulate their expression [122]. 

One of the components of the microprocessor complex, Drosha, shows direct antiviral activity against RNA viruses and engineered chimeric viral genomic RNAs containing human derived pri-miRNA sequences, or well-characterized viral DNA pri-miRNA structures [123,124,125,126]. This activity requires Drosha translocating from the nucleus to the cytoplasm, since most of the replication cycle of RNA viruses is restricted to this compartment [124,125,126]. However, RNA viruses rarely encode microprocessor-dependent miRNAs. Considering that microprocessor cleavage of viral RNAs could impact the steady-state levels of the viral genomes, the potential benefit for RNA viruses to encode miRNAs is still unclear. Efforts to address this issue have shown that, unexpectedly, cleavage of the viral genome by Drosha has a negligible effect on viral fitness [123,127], suggesting that only a small proportion of the total of viral RNA is available for miRNA processing. However, opposite results have been obtained in the context of lentiviral vectors containing microprocessor-dependent pri-miRNA substrates [128]. A recent characterization of Drosha function on natural RNA viruses unveiled that its antiviral activity is independent of RNA cleavage function, and DGCR8 or Dicer factors. Instead, the binding of Drosha to the viral RNA hinders viral RNA-dependent RNA-polymerase activity during replication (Figure 2). This direct antiviral role is not limited to mammalian Drosha and it is suggested to be conserved in other eukaryotes, including fish, plants, and arthropods [129]. 

In contrast, mammalian DNA viruses, such as herpesviruses and polyomaviruses, encode for viral miRNAs, which are essential for successful replication [130,131,132,133,134,135]. Whereas viral-derived miRNAs are produced by the canonical miRNA biogenesis pathway, some viruses have evolved to use alternative biogenesis pathways, such as the integrator complex or tRNAse Z [136,137]. It is unclear how the biogenesis of viral miRNAs affects the abundance and stability of the precursor viral transcripts. DNA viruses restrict the expression of miRNAs to specific viral genes and during certain stages of their lifecycle, such as in latency, a mechanism that may have evolved to avoid a general downregulation of the viral transcripts throughout the whole virus replication cycle. 

### 2.2. Dicer

Double-stranded RNAs (dsRNAs) generated during viral replication can be recognized and cleaved into 21–23 nt-long siRNAs by the cytoplasmic host protein Dicer which are incorporated into the RISC complex and bind complementary viral RNA to induce its degradation. The relevance of RNA interference (RNAi) as an antiviral mechanism in fungi, invertebrates, and plants is well established [138,139,140,141,142], since disruption of any of the critical components of the RNAi response renders these organisms highly susceptible to viral infection. Even though mammals express all the necessary components for RNAi, it has proven difficult to establish whether this pathway acts as a functionally relevant antiviral mechanism. Research into the role of antiviral RNAi in mammalian cells has been confounded by seemingly contradicting results. Deep sequencing experiments on a wide range on viruses failed to detect, or only detected very low levels of virus-derived siRNAs [143,144,145,146,147,148]. Despite these observations, a functional antiviral activity of RNAi in mammalian cells was first presented in the papers by Li and Maillard [149,150] where antiviral siRNAs were detected from both strands of the virus, and blocking the RNAi pathway increased viral replication. 

Classical RNAi organisms are characterized by Dicer gene duplications, with specific and non-interchangeable isoforms specialized in the production of siRNAs or miRNAs [142,151]. For instance, *D. melanogaster* Dicer-2 is specialized in siRNA biogenesis and antiviral defense, whereas Dicer-1 is restricted to miRNA biogenesis [141]. Instead, mammals have retained a single copy of Dicer responsible for both mi- and siRNA biogenesis [73,152]. Comparative functional analyses of Dicer proteins revealed that human Dicer only partially rescues antiviral defense in flies, suggesting that human Dicer is less efficient at cleaving dsRNA [153]. Deletion of the N-terminal helicase domain of human Dicer increases its ability to process dsRNA [154]. Furthermore, a specific Dicer isoform lacking the N-terminal domain is expressed in mouse oocytes, which explains their proficiency to generate siRNAs from both endogenous and exogenous dsRNAs [155]. Similarly, the expression of an N-terminal truncated form of Dicer during viral infections enhances detection of viral derived siRNAs in mammalian cells [146]. All these suggest that mammals have retained certain Dicer antiviral activity. Supporting these findings, mutations in viral factors encoding for suppressors of RNA silencing (VSRs) also improved antiviral siRNA detection, suggesting that viruses have developed mechanisms to counteract RNAi in mammals. Examples of mammalian VSRs include the N protein from coronaviruses, NS1 from Influenza A virus, 3A from human enterovirus 71, and VP35 from Ebola virus, amongst others [148,149,156,157,158].

The impact of Dicer on DNA virus replication is a much-understudied aspect. The non-coding virus-associated (VA) RNAs from adenoviruses are direct Dicer substrates, acting as RNA decoys by competing for Dicer binding to other substrates [159,160]. The VA RNA is necessary to block PKR function, allowing viral mRNA translation [161,162]; Dicer-mediated cleavage of this RNA affect its integrity and as a consequence, its proviral function [163]. 

Disentangling the functional contribution of the RNAi pathway in mammals is complicated by the co-occurrence of the IFN response. Recent observations also suggest that both pathways interact and influence each other.

## 3. Type I IFNs Modulate the Activity of The Small RNA Biogenesis Pathway

The relationship between the IFN response and small RNAs is intricate. For instance, the IFN response is highly regulated by miRNAs, but at the same time, the IFN response regulates miRNA expression and RNAi proficiency to ensure the most efficient antiviral state. 

During homeostasis, miRNAs regulate a large number of genes involved in the IFN response, which suggests that dysregulation of miRNA expression can lead to incorrect levels of these and other cytokines. Importantly, the unbalanced production of IFNs and pro-inflammatory molecules are at the root of human disorders, including autoimmune disease, inflammation, and cancer [164,165]. To ensure correct regulation of the IFN response, miRNAs target genes involved in different stages of the IFN response pathway, including the PRRs, transduction proteins, and transcription factors [166,167,168]. This homeostatic regulation is considered to act as a general dampening down of the IFN response that needs to be de-repressed following its activation. In agreement, in the absence of miRNAs by depletion of *Dicer* in microglia, endometrial, and thymic cells, spontaneous expression of IFNs is observed in the absence of infection [169,170,171]. Remarkably, ISGs also seem to be more significantly regulated by miRNAs than housekeeping genes [172]. Besides this global de-repression, there are also a number of miRNAs whose expression is induced by the IFN response. miR-146 and miR-155 are IFN-induced miRNAs that act as negative feedback loop molecules to shut-down the IFN response [173,174]. These and other miRNAs target components of the IFN response, such as TLR-receptors and the signaling molecules TRAF6, IRAK1, and 2 [175,176], but are also found to directly target cytokines such as TNF-α and IFN-β [177,178].

After transcription, the expression of miRNAs can also be post-transcriptionally regulated by the type I IFN response. Stimulation of cells with the viral mimic dsRNA and activation of IFN expression regulates the processing of pri-miRNAs by the microprocessor. This inhibition leads to a transient depletion of specific miRNAs, which is necessary to robustly express IFN- β and initiate the antiviral response [179]. Along these lines, type I IFNs diminish Dicer expression levels [180] and Dicer cleavage activity [181]. However, the exact mechanism by which the type I IFN response impairs both Drosha and Dicer cleavage activities is still unknown. In the case of the microprocessor, the activation of IFN expression reduces the binding affinity of DGCR8 to its substrates [179]. In the case of Dicer, the RLR LGP2 has been found to compete for Dicer binding to its substrates during the antiviral response [182]. Other factors involved in the recognition of dsRNA, such as ADAR, can also modulate the function of Drosha and Dicer proteins. ADAR1 associates to Dicer to enhance its mi-/si-RNA mediated processing [183], whereas ADAR-mediated editing of pri-miRNAs inhibits both Drosha and Dicer cleavage activity [184,185].

**Figure 3:**
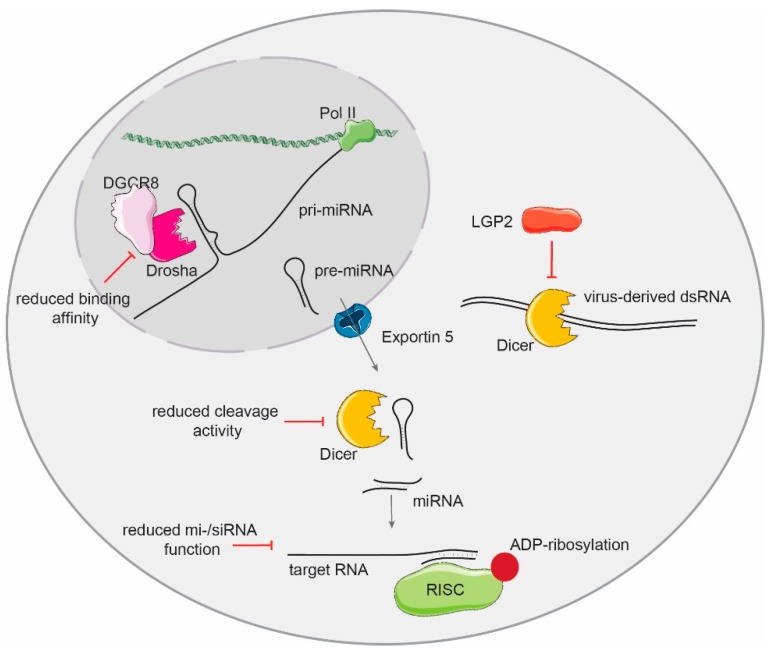
The activation of the type I IFN response impairs the activity and function of the small RNA biogenesis pathway. The microprocessor complex binding affinity and cleavage activity of pri-miRNA substrates is reduced upon IFN expression activation. IFNs also reduce Dicer protein levels and impair its cleavage activity. The function of the RISC complex is also modulated by the IFN response by poly-ADP-ribosylation of the essential component Ago2. The RLR LGP2 interferes with recognition and cleavage activity of Dicer on dsRNAs.

Other parts of the small RNA silencing pathway, such as RISC, are also negatively regulated during IFN activation. Activation of the IFN response induces Ago2 poly-ADP-ribosylation, which correlates with diminished miRNA and siRNA activity in cells during infection [172] (Figure 3).

Other classically-associated factors of the antiviral response can also modulate Dicer activity. PACT, an activator of PKR function [186], and TRBP, an inhibitor of PKR function [187,188], regulate the endonucleolytic activity and accuracy of Dicer cleavage [58,84,87,189,190,191]. More recently, PACT has also been shown to bind and stimulate the ATPase activity of RIG-I like receptors to initiate the antiviral response [192]. Conversely, overexpression of *D. melanogaster* Dicer-2 in human cells blocks IFN-β expression and PKR function [153]. 

A common feature of all the aforementioned factors is their ability to recognize dsRNA [193]. Binding to shared dsRNA molecules may act as a platform for these interactions to occur but also offers an opportunity to compete for binding to the same RNA substrates. Intriguingly, the N-terminal DExD-helicase domain of Dicer shares homology with that of the RIG-I-like family of receptors, highlighting the similarities and evolutionary conservation between the antiviral RNAi and protein-based response in their structural organization [194,195].

Given the growing evidence for a functional incompatibility between these two antiviral systems in mammals, the reason for this becomes more intriguing. Rapid impairment of small RNA biogenesis and RISC function during the activation of the type I IFN response may be necessary for robust ISG expression and establishment of the antiviral state. This is further supported by reports that show mammalian embryonic stem cells and embryonic carcinoma cells have a much better developed RNAi response compared to somatic cells. These cells inherently lack a functional IFN response [196], and are able to process long dsRNA into functional siRNAs [197,198]. Furthermore, RNAi activity in ESCs targeting either endogenous transposable elements [106] or viruses is more pronounced [150]. The shutdown of the IFN response in somatic cells enables the processing of long dsRNA into functional RNAi and inhibits replication of virus with the cognate sequence [199]. However, naturally derived siRNAs can still be detected, to an extent, in IFN-proficient cell lines [158]. All these suggest the presence of a competition between the small RNA biogenesis factors and the type I IFN response when co-occurring in the same cell type. 

## 4. MiRNA-Mediated Regulation of Viruses

Besides regulation of the IFN response, and consequently the antiviral state of cells, miRNAs have been found to directly target viral sequences in human immunodeficiency virus-1 (HIV-1), primate foamy virus-1 (PFV-1), influenza A virus (IAV), hepatitis C virus (HCV), vesicular stomatitis virus (VSV), and the human papilloma virus (HPV) [200,201,202,203,204]. Unlike their endogenous targets, only the minority target the 3′ end of the viral sequences. Although the available experimental data clearly supports a role of miRNAs directly targeting viral genomes or transcripts, its biological significance is still unknown. From an evolutionary point of view, it is difficult to imagine that for very recent infections, such as HIV-1, or low-level infection rates, such as for HCV, there has been enough evolutionary pressure to have developed specific sequences to target these viruses.

Given the importance of miRNAs in the robustness of the IFN response and consequent antiviral state of cells, and the observation of miRNAs directly targeting viral sequences, it is no surprise that there are a number of viruses targeting miRNA regulation. One of the first examples was found in primate foamy virus (PFV-1), which encodes a protein (Tas) that was able to inhibit miRNA function, relieving the suppression of miR-32 [205]. Viral inhibitors of miRNA function are also found in HIV-1 [206,207], Ebola virus [157], Influenza A virus [208], and Vaccinia virus [209], suggesting host miRNAs exert enough pressure to warrant this kind of investment. 

The reverse scenario, where viruses seemingly benefit from endogenous miRNAs, is found in the Flaviviridae family, where HCV uses miR-122 to shield its genome from degradation resulting in increased translation [210,211]. This miRNA is highly expressed in the virus’ natural host cells, where it binds the 5′ UTR of the viral genome, resulting in improved viral replication in an AGO2-dependent fashion [212,213]. Recently, miR-21 was identified as a pro-viral factor for the Zika virus, a member of the same Flaviviridae family as HCV, suggesting that utilizing host miRNAs for their own benefit is common in this virus family [214]. Endogenous miRNAs having pro-viral properties have also been reported during HIV-1 and HCMV infections [215,216].

The observation that miRNAs are important for the regulation and robustness of the IFN response, the direct pro- and antiviral effects of miRNAs and the presence of miRNA-regulating activities of viruses makes it extremely difficult to predict the net effects when manipulating the major miRNA biogenesis factors. Despite this inherent complex network of interactions, in the next section we will have a closer look into the results of manipulating the expression of small RNAs in the context of the consequences on the IFN response and viral dissemination.

## 5. Consequences of Small RNA Biogenesis Manipulation on the IFN and Antiviral Response

### 5.1. Knock-Downs of Small RNA Biogenesis in Somatic Cells

Previous research into the role of small RNAs on the IFN and antiviral response often focused on the manipulation of Dicer protein levels, as this protein is essential for both miRNA and siRNA production (Figure 2). Experiments knocking down Dicer are characterized by a wide variety in outcomes due to a number of factors, such as the level of knock-down, which is determined by the efficiency of the method used, or by the cell line used, which determines both the efficiency of the knock-down, and, more importantly, the inherent immune competence. 

This variability was evidenced in Dicer knock-down experiments in a panel of endometrial cancer lines. Despite similar growth rates, knock-down of Dicer protein levels resulted in a variable increase in cell migration and spontaneous IFN-β expression, which correlated with the accumulation of unprocessed pre-miRNAs in the cytoplasm [169]. This variation likely reflects the involvement of different miRNAs in the regulation of the IFN response and in variations of Dicer knock-down levels combined with cell-specific characteristics. This paper also showed that knocking down Dicer by shRNAs might be efficient, but not sufficient to completely impair miRNA biogenesis. Surprisingly, knock-down of Drosha in the same cell lines did not result in IFN-β induction, suggesting that accumulation of unprocessed pre-miRNAs in the cytoplasm might accidently trigger the IFN response [169]. The same was found in HEK293 cells where the knock-down of Dicer, but not Drosha, resulted in the upregulation of IFN and accumulation of dsRNA, probably from retrotransposon origin [217]. 

A similar approach in Vero cells, which have an impaired IFN response, showed that Dicer knock-down had a limited effect on cell growth, but did result in an increased replication of influenza A virus (IAV) [218]. Interestingly, IAV infection results in the downregulation of Dicer protein levels, and the IAV NS1 protein has also been shown to interfere with siRNA function, suggesting that IAV actively modulates small RNA expression for its own benefit [218,219,220]. Targeting the miRNA pathway is also observed during vaccinia virus (VV) infections, where Dicer is cleaved by the viral protease i7 to induce its degradation. Interestingly, neither the knock-down of Dicer by siRNA nor overexpression improves VV replication, suggesting that modulation of miRNA expression is only beneficial for VV replication when timed correctly [221].

### 5.2. Knock-Out of Small RNA Biogenesis in Somatic Cells

The knock-down of small RNA biogenesis results in an impairment, but not a total abrogation of small RNA function, therefore masking the true effect of the absence of small RNAs on the IFN response and viral outcome. The use of complete knock-outs has been hampered by the lethal phenotype associated with Dicer ablation, highlighting the essential role for miRNAs in cell survival [222]. 

The effect of miRNA deficiency on the IFN response and viral replication was assessed in mice where Dicer function was disrupted (hypomorphic allele), but not completely abrogated [223]. Interestingly, infections with five different viruses showed no differences in viral replication, only a significant increase in vesicular stomatitis virus (VSV) and herpes simplex virus replication. Further investigation found *Dicer*-deficient mice to be hypersensitive to VSV infection due to the absence of specific miRNAs directly targeting the VSV genome, but not to changes in the IFN response [223]. In a later report, a direct link between the IFN response and loss of miRNA regulation was found using the same mouse strain and herpesvirus infection. *Dicer* hypomorph mice and isolated macrophages were hypersensitive to mouse Cytomegalovirus (mCMV) and despite the upregulation of IFN dependent genes, the ISG response was impaired after infection. The authors concluded that a rapid and transient de-repression of the miRNA regulated IFN response genes is necessary for a functional antiviral response [224]. This was further confirmed in our recent publication, where a short-lived downregulation of specific miRNAs was observed during the activation of the IFN response and was found to be necessary for robust IFN expression and consequent antiviral activity [179].

To circumvent the lethal phenotype of a complete *Dicer* knock-out in somatic cells, a viral inhibitor of RISC function, vaccinia virus protein VP55, was used as an alternative. VSV was engineered to express the VP55 protein and used to test the role of small RNAs in antiviral defense. A reduction in VSV replication was found and attributed to an increased expression of the host antiviral response. This was supported by the observation that the same infections in IFN-negative mice nullified the difference in replication [143,209]. It should be noted that this result was obtained from in vivo infections in mice but could not be recapitulated in vitro with fibroblasts and bone marrow-derived macrophages [143]. Disabling miRNA function for prolonged periods of time by overexpression of VP55 resulted in an increase of pro-inflammatory cytokines expression, without impacting the early responses to virus infections [225]. 

Despite the effect on the viability of impaired Dicer expression in cells and other model organisms, a viable homozygous knock-out of *Dicer* was obtained in HEK293T cells [226]. Using this model, it was observed that the absence of *Dicer* does not affect the replication of a large number of viruses, including VSV [144]. An interesting addition to this observation is that knocking out Drosha in the same cells where Dicer is absent resulted in increased sensitivity to positive stranded RNA viruses (+ssRNA), despite higher expression of antiviral genes, such as *IFIT1*. A direct antiviral role for Drosha was proposed, in which binding of this factor to viral hairpin structures blocks polymerase access during replication [129].

A consistently understudied issue when assessing the function of Dicer and Drosha during viral infections is the impact of the absence of miRNAs on other cellular processes outside the antiviral response. Knocking out *Dicer* in HEK293T cells results in a considerable reduction in proliferation [226], a relevant observation for these experiments since the availability and speed of the cellular machinery may have a major impact on viral replication. Furthermore, manipulations of small RNAs are also likely to influence the expression of cellular receptors and entry mechanisms on which viruses rely to initiate infections. Conclusions driven by these cellular models will benefit from relatively simple controls, such as binding and entry assays, when comparing viral infections in these profoundly different cell lines.

### 5.3. Small RNAs and IFN Response in Embryonic Stem Cells

Similar experiments in embryonic stem cells (ESCs) have addressed the role of small RNAs and their biogenesis factors during the antiviral response. ESCs have the unique ability to indefinitely self-renew and give raise to any tissue of the adult organism. Early stages of development, including pre-implantation blastocysts from which ESCs are derived, suppress the type I IFN response, suggesting an incompatibility between pluripotency and this antiviral pathway [196,227]. Another interesting aspect of ESCs is that, unlike somatic cells, they are able to cleave and process viral derived long perfect dsRNA using Dicer [154,155,228]. As observed in somatic cells, the loss of *Dicer* in ESCs results in a delayed cell cycle progression and an increase in apoptosis [228]. These are accompanied by a significant de-repression of retrotransposable element expression and an increased expression of genes involved in immunological responses [229,230]. Recent findings by our group have confirmed that the disruption of miRNAs in ESCs has huge consequences for the cell’s innate immune responses. The disruption of either *Dicer* or *Dgcr8* expression in mouse ESCs leads to a decrease in susceptibility to TMEV and IAV infections. Interestingly, the decrease in susceptibility was larger in the absence of *Dgcr8* compared to *Dicer*, corroborating that Dicer might have additional antiviral roles, as previously suggested in the antiviral RNAi response [150]. Knocking out miRNA function provided ESCs with the ability to activate the IFN response by increasing the expression of the central factor for RNA immunity, MAVS [231]. Therefore, miRNAs, and more specifically, miR-673, was suggested to be central to silence the type I IFN response during pluripotency.

## 6. Conclusions

Studies investigating the role of small RNAs and the innate immune response to viral infections have uncovered a complex network of interactions. The overall effect of impairing small RNA biogenesis on the host’s antiviral response is difficult to predict, as small RNAs are responsible for silencing the IFN response, but at the same time are required to establish a robust antiviral state. Another layer of complexity is added by the observation that some miRNAs can directly target viral sequences, whereas others increase viral fitness. Integrating all these processes will be the key in providing a comprehensive understanding of the relationship between small RNAs and the type I IFN response. 

## Figures and Tables

**Table 1:** Pattern recognition receptors (PRRs) implicated in nucleic acid sensing. Different classes of PRRs operate in distinct cellular compartments and recognize ligands that are absent or rare in the host.

Pattern Recognition Receptors (PRRs)		Location	Nucleic Acid Ligand	Signaling Adaptor
Toll like receptors	TLR3	Endosome	dsRNA	TRIF
	TLR7/8	Endosome	GU-rich ssRNA	MyD88
	TLR9	Endosome	Unmethylated CpG DNA	MyD88
RIG-I like receptors	RIG-I	Cytoplasm	5′ppp RNA, short dsRNA	MAVS
	MDA5	Cytoplasm	Long dsRNA	MAVS
	LGP2	Cytoplasm	Termini of dsRNA	
Others	cGAS	Cytoplasm	dsDNA	STING
	PKR	Cytoplasm	dsRNA	eIF2α
